# Contribution of Drugs to Drowning in Scotland from 1996 to 2020

**DOI:** 10.2174/1570159X20666220830110758

**Published:** 2023-09-01

**Authors:** John Martin Corkery, Giovanni Martinotti, Fabrizio Schifano

**Affiliations:** 1 Psychopharmacology, Drug Misuse and Novel Psychoactive Substances Research Unit, School of Life and Medical Sciences, University of Hertfordshire, College Lane Campus, Hatfield, AL10 9AB, United Kingdom

**Keywords:** Drowning, Scotland, drug-related, intoxication, fatal, benzodiazepines

## Abstract

**Objective:**

Psychoactive substance use (including alcohol) can affect risk perception, leading to accidents and deaths. There is little detailed or up-to-date information on the role of drugs in drownings in the United Kingdom (UK). This Scottish case-study aimed to fill this knowledge gap.

**Methods:**

Anonymised data for individual drug-poisoning-related drowning registered from 1996 to 2020 were provided by the National Records of Scotland. Statistical analyses were performed for socio-demographics, ICD coding, cause of death, and substances implicated.

**Results:**

It has been reported that death registrations increased from 7 in 2017 to over 20 during 2019-20. These deaths (n=160) accounted for <1% of all drug-related poisoning deaths; this proportion rose to record levels (c.1.5%) during 2019-20. Most deaths (69%) involved males. The mean age was 39.8 (range 16-81, SD 15.0) years. The main drug classes implicated were: opiates/opioids (41%), benzodiazepines (31%), stimulants (19%), and antidepressants (14%). Moreover, 57% of benzodiazepines were ‘designer’ drugs.

**Conclusion:**

Scottish drownings associated with drug consumption are increasing rapidly. It has been observed that central nervous system depressant drugs (*e.g.*, opioids, benzodiazepines, alcohol) are often involved in drowning. ‘Designer’ benzodiazepines are a principal factor in increasing Scottish drug-related poisoning deaths; they may be partially responsible for increasing numbers of related drownings. Evidence-based strategies to further reduce the number of preventable drownings should include reference to the dangers of drugs.

## 1 INTRODUCTION

Intoxication due to alcohol and/or drugs can, in some instances, leads to accidental deaths. Although most of these are associated with road traffic accidents (RTAs) or workplace accidents, they can occur in other situations [1]. In the United Kingdom (UK), based on deaths reported to the National Programme on Substance Abuse Deaths (NPSAD) in 1999, Ghodse *et al.* [2] noted that about 1% of these deaths were a result of asphyxiation, accidental drowning or multiple injuries. Moreover, using NPSAD, but with a study panel of coroners in England, Oyefeso *et al.* [3] examined the nature, extent and pattern of fatal injuries under the influence of psychoactive drugs (FIUI) from January, 1999 to December, 2001. The principal mechanism for intentional FIUI is suffocation, while the predominant mechanisms in unintentional FIUI are RTAs and falls. The lead author and colleagues have also commented on deaths due to exposure and drowning following the consumption of recreational drugs, including ecstasy (MDMA) [4], GHB/GBL [5], ketamine [6, 7], methoxetamine [8], mephedrone [9, 10]. The common causal element in such instances is that intoxication impairs judgment, including the perception of risk.

Globally, after road injuries, falls, and inter-personal violence, drowning has the fourth highest mortality rate for injury [11]. Approximately 360,000 deaths are reported annually from unintentional drowning [12, 13]. The Royal Life Saving Society UK (RLSS UK) reports that, on average,73 individuals die each year due to a substance-related drowning; this is more than one-quarter of all accidental drownings [14]. The latest statistics from the National Water Safety Forum’s (NWSF) WAter Incident Database (WAID) indicate that there were 69 drownings in 2020 with a reported presence of alcohol and/or drugs (where known) across the UK [15]. Thirty deaths involved persons aged under 35 years. The corresponding figure in 2015 was 65, of which 55 had alcohol detected, three with alcohol and drugs, and seven with only drugs [16].

A range of recreational drugs has been implicated in UK drownings, including ecstasy (MDMA), GHB/GBL, ketamine, methoxetamine, and mephedrone. Overseas evidence suggests that psychotropic drugs may play a significant role in drowning, whether on their own or in conjunction with alcohol, particularly due to their effects on cognition and psychomotor function [17].

A Finnish study on unintentional drownings found that 7.9% of cases involved psychotropic drugs alone, with a further 18.4% involving such drugs in combination with alcohol [17]. A Swedish study reported that 13% of drownings were positive for alcohol and psychoactive drugs; the rate is lower (9%) for unintentional cases than intentional and cases of undetermined intent (both16%) [18]. A study in the United States (US) on drowning found that only 3% of accidental cases were positive for illicit drugs and a further 7% for both ethanol and illicit drugs; only 3% of suicides were positive for illicit drugs, with no cases positive for ethanol and illicit drugs; there were no cases of undetermined intent positive for illicit drugs, with or without ethanol [19].

## 2 STUDY RATIONALE AND AIMS

The only public information currently available regarding the involvement of psychoactive drugs in UK drownings is that from the NWSF [16]. However, neither the NWSF nor RLSS UK has published any recent information on this topic. A recent audit of UK drowning from 2012 to 2019 indicated that 16% (n=820) of all cases involved drugs and/or alcohol; of which, alcohol alone accounted for 74%, alcohol with drugs 14%, and drugs alone 13% [20].

The Home Office Scientific Advisory Branch established a pilot study in 1974 to improve official statistics on drowning. The study period covered 1975-7, and some statistics were published [21, 22]. Although the Home Office did continue to publish statistics on drownings covering the period up to 1980, the only psychoactive substance looked at in terms of acting as a contributory factor was alcohol [23]. Furthermore, information only covered England and Wales.

The Royal Society for the Prevention of Accidents (RoSPA) compiled and published annual statistics on UK drownings from 1983 to 2002; however, these varied in content and format. Alcohol was only mentioned in 1999-2002 figures; there was nothing about drug consumption. One interesting aspect in respect of activities/behaviours in which decedents were engaged prior to drowning was mentioned in 1983 that 9 decedents were engaged in ‘glue-sniffing’ related activities, *i.e.*, volatile substance abuse (VSA); however, there was no mention of such incidents in later years. Unfortunately, there are no specific mentions of drownings in the causes of death given in the UK published statistics on VSA-related deaths [24, 25] or those published by the Office for National Statistics (ONS) for England and Wales [26]. This cause of death would have been included in the ‘other’ category (personal knowledge of the lead author in compiling these statistics). The only way to triangulate the findings in the 1983 RoSPA statistics would be to access the archived VSA deaths database and undertake an ad hoc investigation.

Thus, there is a lack of detailed information published concerning the contribution, if any, of psychoactive substances to UK drowning fatalities. The main aim of this study, therefore, was to provide more up-to-date information on such events, using Scotland as a case-study. The objectives were to document the socio-demographics of those drowning, the drugs involved and their role(s) in death, the manner and cause of death, and patterns over time.

## 3 DATA SOURCES AND METHODS

The lead author has access to anonymised case-level data on drug-related poisoning deaths registered by the National Records of Scotland (NRS). This access has been granted as part of a European Union-funded research project (EU-MADNESS) led by the University of Hertfordshire. Data currently accessible covered deaths registered between January 1^st^, 2013 and December 31^st^, 2020. Additional data for 1996-2012 were extracted by NRS using the same filters used for the later period and provided to the lead author.

The datasets contain information on the following variables: details of registration, including year; month and year of death; sex; age at death; ICD-10 codes [27], including underlying cause of death; cause of death text; list of ‘poisons’ considered by the pathologist to have caused or contributed to death, and other substances found in post-mortem toxicology. Relevant cases were selected by text searches of the cause of death for ‘drowning’, ‘immersion’ or ‘submersion’. There was little difference between the year of death occurrence and the year when the event was registered.

Statistical analyses were primarily performed using MicroSoft^®^ Excel^®^ (version 10), including frequencies, proportions, and descriptive statistics. The Chi-square test was used to compare proportions for statistical significance, and the t-test to compare mean ages.

Ethical approval is not required in the UK for studies whose subjects are deceased and solely involve retrospective reviews of death records.

## 4 RESULTS

Overall, 162 such cases had ‘drowning’ or ‘immersion’ mentioned in the ‘cause of death’ field(s) on the death certificates; there were no mentions of ‘submersion’. Two cases were excluded as they related to drownings in a slurry pit [28], and no specific substance was noted by the pathologist as having caused or contributed to the incident [29]. This left 160 cases for analysis.

The NRS drug-poisoning database covers the period from 1996 onwards; the first case meeting the inclusion criteria occurred in 1997. The number of deaths each year is presented in Fig. (**1**). Annual variations were reported between 1997 and 2017, with minor peaks in several years. However, there was a rapid increase in recent years, from 7 deaths in 2017 to 12 in 2018 to over 20 in 2019 and 2020. Possible explanations for this pattern are examined below.

These drownings, on average, account for less than 1% (mean = 0.875, Std Dev 0.38) of all drug-related poisoning deaths in the period from 1996 to 2020; this proportion ranges from 0.000% to 1.574% (Table **1**). In 2019 and 2020, the proportions increased four- or five-fold, against a background of all drug-related deaths doubling.

**Fig. (2):**
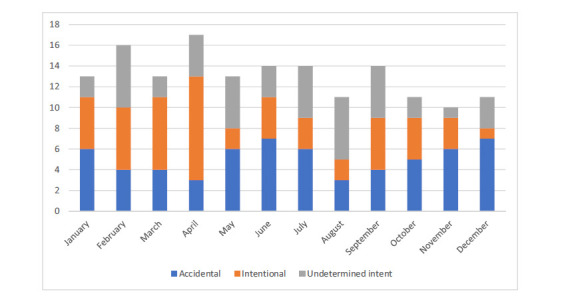
Number of drug-related poisoning deaths involving drowning by month of .occurrence and intent in Scotland from 1996 to 2020.

There is no seasonality in terms of months when deaths occur; the number per month ranges from 10 to 19, with two peaks in February and April (Fig. **2**).

Nearly seven-tenths (n = 110; 68.75%) of decedents were male. The mean age of those drowning was 39.84 (range 16-81, Std Dev = 15.02) years: males tended to be younger (mean 38.12, range 16-80 years, Std Dev = 14.45) than females (mean 43.64, range 21-81 years, Std Dev. = 15.70). This difference in mean age is statistically significant (t-test, *P* = 0.0308).

The most common substances implicated were: drugs alone or in combination (59%), drugs and alcohol (31%). There were 15 cases where no specific substance was mentioned in the cause of death, but 12 of these had ‘drug abuse/drug misuse’ mentioned as a contributory cause. The maximum number of substances implicated was 7, and the mean was 1.97.

A number of different classes of the drug were deemed implicated in death (Table **2**). The main ones were (in descending order): opiates/opioids (41%), benzodiazepines (31%), stimulants (19%), and antidepressants (14%). There were 15 cases with ‘unspecified drugs’. No volatile substances were specified.

The commonest combinations of drug classes implicated are presented in Table **3**. The main ones were: opiates/opioids + benzodiazepines (28), benzodiazepines + stimulants (11), benzodiazepines + antidepressants (7), opiates/opioids + stimulants (6), opiates/opioids + antidepressants (5). Alcohol was implicated in 50 deaths, most commonly with the following drug classes: alcohol + benzodiazepines (20), alcohol + opiates/opioids (19), and alcohol + stimulants (16); these groupings are not mutually exclusive. At least one central nervous system depressant (alcohol, benzodiazepine, diphenhydramine, opiate/opioid) was implicated in 106/160 (66.25%) deaths. It is of note that the only novel psychoactive substances (NPS) implicated in these drownings were benzodiazepines. Indeed, ‘designer benzodiazepines’ accounted for 57% of drownings involving this drug class (Table **3**).

Based on ICD-9 [30] and ICD-10 codes [27], just under two-fifths (n = 61; 38.2%) of all deaths were accidental in terms of intent, whereas about one-third (n = 52; 32.5%) were intentional and just over one-quarter (n = 44; 27.5%) were of undetermined intent. Two were deemed a homicide and one natural cause. Table **4** contains details of the initial (underlying) event leading to death.

Little information can be gleaned from the ‘cause of death’ field and/or the ICD-10 codes used with respect to the circumstances of the death. ‘Drug abuse’, ‘drug abuser’ or ‘drug misuse’ was mentioned in 65 (41%) of the cases. In the majority of cases (n = 129; 81%), the type of water was unspecified; in 16 cases, the body of water was simply referred to as ‘natural water’, and there were four instances of ‘fresh water’. However, “bath” was mentioned nine times and “cold water” twice. The terms ‘cold’ and ‘hypothermia’ were mentioned in a total of six cases, as were ‘falls’ or ‘jump from height’. To get a better insight into such issues, it would be necessary to undertake record linkages, such as by NPSAD and the Scottish National Drug-Related Deaths Database [31].

For the 110 males, the most common intent was accidental (44%), followed by undetermined intent (29%) and intentional (25%), along with one homicide and one natural causes. For the 50 females, the most common intents were: intentional (50%), accidental (24%), and undetermined intent (24%); there was one homicide. Of the 67 deaths in those aged under 35 years, accidental accounted for 33% of cases, undetermined intent for 37%, intentional for 28%, and there was one homicide. For the 93 cases aged 35 years or more, the most common intent was accidental (42%), followed by intentional (35%), and undetermined (20%); there was one homicide and one case of natural causes.

## 5 DISCUSSION

This section attempts to make comparisons with other UK-based resources and the international literature, bearing in mind the nature of the types of data available and variables recorded.

### 5.1 Socio-demographics

According to the WAID dashboard [32], males accounted for 87% of drowning in Scotland from 2014 to 20. Most drownings during 2018-20 occurred in the over 35 years age group. In this study, the proportion of males was somewhat lower (69%), but the mean age of about 40 years was in line with the WAID data. Using the published ‘drug misuse’ poisoning figures (as those using the wider ONS definition are not published), during 1996-2020, males accounted for 82.66% of such deaths in Scotland, whilst in 2000-20 those aged 15-34 years accounted for 4,886 deaths compared to 7,977 in the 35-64-year age-groups. Thus, the age-related patterns in drownings associated with drug-related poisoning are broadly in line with drug-poisoning deaths [33].

### 5.2 Intentionality

An examination of data compiled by the NWSF available from their WAID annual reports (https://www.national watersafety.org.uk/waid/annual-reports-and-data) indicated that during 2015-20, there were 520 drownings (of any type) in Scotland. During the same timeframe, data in Fig. (**1**) indicate that there were 78 drug poisoning deaths involving drowning or immersion, accounting for 15% of all drownings.

Of the 520 drownings reported by the NWSF in 2015-20, 41.7% were suspected accidents, and 24.2% were suspected suicides; however, the type of drowning was not recorded in 22.9% of cases. The remaining types were attributed to suspected crime (1.0%) and natural causes (1.3%). The NRS-derived proportions for drug-related poisoning drownings were: accidental 38.2%, intentional 32.5%, and undetermined intent 27.5%. These reflect the overall NWSF pattern, but with a higher proportion deemed accidental. Using the Chi-square test, the difference between proportions for accidental deaths is not statistically significant (*P* = 0.4353) or not recorded/undetermined (*P* = 0.2337) but is for suicide/intentional deaths (*P* = 0.0368).

An earlier study covering 10,092 suicide and undetermined death by drowning in England and Wales 1979-2001 concluded that combining such causes (62% open vs. 38% suicide) may lead to over-estimated rates and misleading trends in suicidal drownings [34]. Whilst these observations were made in respect of a period when deaths were classified using ICD-9 [30], this is still an important consideration at the present time. Although that study did not examine the involvement of substances in drowning, some useful observations could be extracted from the results. Over the period as a whole, suicide verdicts were more likely to be handed down in drownings involving older compared to younger age groups; women, especially older ones, were more likely than men to receive a verdict of suicide. The findings in our study are in line with these results: 35.5% of those aged 35+ years received an ‘intentional’ coding compared to 28.4% of those <35 years, whilst those <35 years were more likely to be ascribed an ‘undetermined intent’ (37.3% *vs*. 20.4%); females (50.0%) were more likely to receive an ‘intentional’ coding than males (24.6%) but less likely (24.0%) to be ascribed an ‘undetermined intent’ than males (29.1%).

### 5.3 Role of Drugs

The Water Incident Dashboard instigated by the National Fire Chiefs Council (NFCC) indicates that, in 2020, intoxication was implicated in 29.13% of UK drownings: alcohol 19.29%, alcohol and drugs 6.30%, and drugs for 3.54% [35]. In our study, the respective proportion for drugs and alcohol was 9/23 (39.13%) and that for drugs only was 12/23 (52.17%) in 2020. These are the reverse order of contributions of the NFCC figures. The presence of alcohol and/or drugs was reported in about one-third of fatal drownings in Wales in 2017 and 2018 [36].

An examination of the role of psychotropic drugs in unintentional Finnish drownings found that the commonest substances were benzodiazepines, antidepressants, and ‘Z’ drugs [17]. A Swedish study reported that 22% of unintentional drownings were positive for pharmaceutical drugs and 9% for illicit drugs; by comparison, the relevant proportions for intentional drownings were 12% and 15%, whilst those of undetermined intent were 44% and 15% [18]. The findings of these studies echo the involvement of these medications in the present study. Nearly one-third (31%) of accidental drownings in a US study were positive for drugs; unfortunately, the classes of drugs were largely unspecified, although it was noted that the most common illicit drugs detected were cannabinoids and cocaine or its metabolites [19]. However, positivity for drugs does not necessarily imply causation or implication, and this aspect was not specifically addressed by the latter two studies.

NPS or ‘designer’ benzodiazepines are a principal contributing factor to the increase in drug-related poisoning deaths observed in Scotland during the last few years [37] and may be responsible, partly, for the increased numbers of drug-poisoning-related drownings. Table **1** shows that the highest proportions of drug-related poisoning deaths involving drowning to date were for registrations in 2019 and 2020, a period when the involvement of this class of drug in drownings was highest. This aspect merits further investigation, especially in light of similar findings regarding the involvement of prescription benzodiazepines generally in Finnish drownings [17].

### 5.4 Seasonality

There is little variation in the number of drownings across the months (Fig. **2**). This contrasts with the pattern observed by Salib and Agnew [34], a peak in March followed by a decline to June, followed by a period of stability to a lower peak in January. The WAID dashboard [32] indicates that for accidental drownings in Scotland during 2014-20, there is an increase from May to August with a peak in July; this is much more in line with what one would expect in relation to warmer weather and access to natural water during the summer months. However, as many drownings considered here are also of intentional or undetermined intent, the pattern for accidental deaths may be obscured. Fig. (**2**) indicates that the distribution for accidental deaths is bimodal, with peaks in June and December. By contrast, intentional deaths peaked in the spring, and for deaths of undetermined intent, there was no discernible pattern.

It was previously found, through examination of coroners’ records, that drowning fatalities following consumption of drugs (with or without alcohol) occur both in the home (in a bathtub) and in ‘natural water’, whether flowing (sea, rivers, canals, and streams) or static (reservoirs, swimming pools, lakes, and ponds). In the home environment, individuals have become sleepy or lost consciousness under the effects of drugs, many of which have sedating properties, and their heads have subsequently slipped under the water, cutting off their oxygen supply, and leading to death. In terms of ‘natural water’, the typical scenario is that of an individual under the influence of drugs (and alcohol) who decides to go for a swim without appreciating the risks involved. These risks might include the presence of fast currents and undertows, coldness of the water, their own lack of swimming ability, undertaking these activities at night/in the dark, *etc.* Exposure to cold temperatures is common in drownings but also in deaths that have occurred as a result of individuals becoming disorientated due to the effects of drugs (and alcohol), getting lost, finding themselves without shelter and falling asleep with fatal consequences, *e.g.*, hypothermia.

### 5.5 Implications for Drowning Prevention Strategies

There were only a few cases where the circumstances, such as cold or hypothermia, were captured in drowning cases of drug-related poisoning deaths on death certificates. Such information is captured on the UK WAID database, but its usefulness is limited by the lack of information on the involvement of drugs (as opposed to alcohol) in such fatalities. The limitations of the WAID database have been partially mitigated by the NFCC’s database. However, to get more detailed information and thereby provide more evidence-based advice on reducing drowning associated with drug use, it is recommended that stronger record linkages are made with those formally investigating such incidents, *i.e.*, procurator fiscals in Scotland, and coroners and medical examiners elsewhere in the UK.

Whilst the UK experienced a substantial fall in all-age mortality counts for unintentional drownings from 488 in 1990 to 335 in 2017 [38], more can be done to further reduce this preventable cause of death, especially where psychoactive substances play a role.

Although the UK drowning prevention strategy [39] mentions alcohol in relation to behavioural factors contributing to drowning, there is no mention of drug consumption. The same is true for the Scottish strategy [40]. However, the Welsh strategy notes: “While alcohol and/or drugs were not directly attributable in all cases, it is widely accepted and regularly reported by experts that alcohol is a drowning risk factor” [36].

A stronger message needs to be disseminated about the danger that drugs on their own and in combination with alcohol can have in terms of risk perception. This message needs to be aimed not just at those who are drug-dependent but also at recreational consumers. Prescribers should also consider the dangers of drowning for their patients who are prescribed or use psychoactive substances and who like to participate in swimming or other water-based activities, especially individuals who are most likely to drink alcohol [17].

## 6 STUDY STRENGTHS AND LIMITATIONS

The main strength of this study is that it is believed to be the first substantive and in-depth investigation into UK drownings associated with drug-related poisoning deaths. Furthermore, the period covered is 25 years (1996-2020).

The total number of cases is only 160. Therefore, the findings may not be representative of UK drug-related poisoning deaths by drowning.

There is a lack of information captured by death certificates regarding the circumstances leading up to and including death, past medical and psychiatric history, prescription medication history, substance misuse history, *etc.* Such details could be derived from either record linkage studies and/ or psychological autopsies.

However, the information presented here does serve to provide a starting point for a larger-scale study in terms of geographical and temporal dimensions, as well as more in-depth investigations.

## CONCLUSION

There is little detailed information available in the UK and elsewhere on the potential and actual fatal role of drug consumption (on its own or in combination with alcohol) in causing and contributing to drownings.

This study provides the first substantial evidence of this phenomenon. It can provide the basis for evidence-based strategies to further reduce the number of preventable drownings that occur. It is recommended that those responsible for drawing up and implementing such strategies commission detailed research at a national or regional level to explore the issues outlined above. Educational materials need to include references not only to the dangers of alcohol in relation to drowning but to drugs.

The data on which these findings are based are limited by the nature and purpose for which they are gathered. However, they provide a basis for future studies, which should include record linkage and psychological autopsy approaches.

Drownings associated with drug consumption appear to be increasing in Scotland in recent times; this situation warrants monitoring and further investigation.

## Figures and Tables

**Fig. (1):**
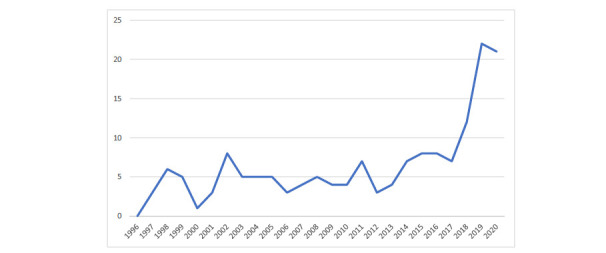
Number of drug-related poisoning deaths involving drowning by year of occurrence in Scotland from 1996 to 2020.

**Table 1:** Number and proportions of all drug-related poisoning deaths accounted for by drownings in Scotland by year of registration from 1996 to 2020.

**Year**	Number of Drug-related Poisoning Deaths*	**Of Which, Drownings**	**Proportion (%) of of all Srug-related Poisoning Deaths Accounted for by Drownings**
1996	460	0	0.000
1997	447	3	0.671
1998	449	6	1.336
1999	492	5	1.016
2000	495	1	0.202
2001	551	3	0.544
2002	566	8	1.413
2003	493	5	1.014
2004	546	5	0.916
2005	480	5	1.042
2006	577	3	0.520
2007	630	4	0.635
2008	737	5	0.678
2009	716	4	0.559
2010	692	4	0.578
2011	749	7	0.935
2012	734	3	0.409
2013	685	4	0.584
2014	743	7	0.942
2015	813	8	0.984
2016	997	8	0.802
2017	1,045	7	0.670
2018	1,313	11	0.838
2019	1,406	21	1.494
2020	1,461	23	1.574
1996 - 2020	18,277	160	-
Mean	731.08	6.40	0.875
Range	447 - 1461	0 - 23	0.000 - 1.574
Std. Dev.	295.91	5.28	0.38

*Using the wide ONS definition rather than the ‘Drug Misuse’ definition, which is restricted to drugs controlled under the Misuse of Drugs Act 1971.

**Table 2:** Main substance classes and selected substances implicated in drug-related poisoning deaths involving drowning in Scotland from 1996 to 2020.

**Substance(s)**	**Number**	**%**
*N*	160	100.0
Opiates/opioids	65	40.6
of which,	-	-
Buprenorphine	3	1.9
Codeine	6	3.8
Dextropropoxyphene/propoxyphene	2	1.3
Dihydrocodeine	12	7.5
Fentanyl	1	0.6
Heroin/diamorphine	21	13.1
Hydrocodone	1	0.6
Methadone	24	15.0
Morphine	17	10.6
Tramadol	7	4.4
Benzodiazepines	49	30.6
of which,	-	-
Diazepam	19	11.9
Temazepam	5	3.1
Alprazolam	2	1.3
of which, the following were Novel Psychoactive Substances	28	17.5
Etizolam	23	14.4
Diclazepam	1	0.6
Flualprazolam	1	0.6
Flubromazepam	1	0.6
Flubromazolam	1	0.6
Phenazepam	1	0.6
Stimulants	30	18.8
of which,	-	-
Amphetamine	7	4.4
Cocaine	19	11.9
MDMA/Ecstasy	6	3.8
Antidepressants	23	14.4
of which,	-	-
Amitriptyline/nortriptyline	4	2.5
Citalopram	2	1.3
Clomipramine	1	0.6
Dothiepin	1	0.6
Doxepin	1	0.6
Fluoxetine	4	2.5
Sertraline	3	1.9
Venlafaxine	5	3.1
‘Z’ drugs (zolpidem, zopiclone)	6	3.8
Antipsychotics	5	3.1
Gabapentinoids	5	3.1
Antiepileptics	2	1.3
Antihistamines	2	1.3
‘Unspecified drugs’	15	9.4
Alcohol	50	31.3

**Table 3:** Main combinations of substances implicated in drug-related poisoning deaths involving drowning in Scotland from 1996 to 2020.

**Combinations of Substances**	***Number***	***%***
*N*	160	100.0
*Drug class combinations*	-	-
opiates/opioids + benzodiazepines	28	17.5
benzodiazepines + stimulants	11	6.9
benzodiazepines + antidepressants	7	4.4
opiates/opioids + stimulants	6	3.8
opiates/opioids + antidepressants	5	3.1
*Alcohol in combination with drugs*	-	-
Alcohol + benzodiazepines	20	12.5
Alcohol + opiates/opioids	19	11.9
Alcohol + stimulants	16	10.0
Alcohol + opiates/opioids + benzodiazepines	7	4.4
Alcohol + benzodiazepines + stimulants	4	2.5
Alcohol + opiates/opioids + stimulants	2	1.3

**Table 4:** Drug-related poisoning deaths involving drowning by underlying cause in Scotland from 1996 to 2020.

**Cause of Death**	**Number**	**%**
Drowning intentional	33	20.63
Drowning of undetermined intent	26	16.25
Accidental poisoning	20	12.50
Unspecified drowning	18	11.25
Drowning in natural water	16	10.00
Intentional poisoning	13	8.13
Poisoning of undetermined intent	11	6.88
Mental and behavioural disorders due to use of opioids/cocaine- dependence syndrome	4	2.50
Suicide and self-inflicted injury by submersion [drowning]	4	2.50
Immersion unspecified	3	1.88
Assault by drowning/submersion	2	1.25
Jump from height intentional	2	1.25
Submersion [drowning], undetermined whether accidentally or purposely inflicted	2	1.25
Car occupant [any] injured in a non-collision transport accident	1	0.63
Drowning following fall into fresh water	1	0.63
Exposure to unspecified factors causing other and unspecified injury	1	0.63
Ischaemic heart disease	1	0.63
Jump from a height of undetermined intent	1	0.63
Mental and behavioural disorders due to multiple drug use and use of other psychoactive substances	1	0.63
*N*	160	-

## Data Availability

The data will not be made available as they are subject to an agreement between the National Records of Scotland and the authors’ institution.

## LIST OF ABBREVIATIONS

EU-MADNESS: EUropean-wide, Monitoring, Analysis and knowledge Dissemination on Novel/Emerging Psychoactives
FIUI: Fatal Injuries Under the Influence of Psychoactive Drugs
GBL: Gamma-Butyrolactone
GHB: Gamma-Hydroxybutyric Acid
ICD: International Classification of Diseases
MDMA: 3,4-Methyl-enedioxy-methamphetamine
NFCC: National Fire Chiefs Council
NPS: Novel Psychoactive Substance
NPSAD: National Programme on Substance Abuse Deaths
NRS: National Records of Scotland
NWSF: National Water Safety Forum
ONS: Office for National Statistics
RLSS: Royal Life Saving Society
RoSPA: Royal Society for the Prevention of Accidents
RTA: Road Traffic Accident
Std Dev: Standard Deviation
UK: United Kingdom
VSA: Volatile Substance Abuse
WAID: Water Incident Database
